# Complete genome sequences of 34 Arctic marine bacteria

**DOI:** 10.1128/mra.00158-25

**Published:** 2025-08-25

**Authors:** Michael C. Sadler, Sayaka Mino, Robert M. Morris

**Affiliations:** 1 School of Oceanography, University of Washington 7284, Seattle, Washington, USA; 2 Laboratory of Microbiology, Faculty of Fisheries Science, Hokkaido University 68275 , Hakodate, Japan; California State University San Marcos, San Marcos, California, USA

**Keywords:** Arctic, SAR11, SUP05, SAR116, nanopore

## Abstract

We report the complete genome sequences of 34 Arctic marine bacteria, including 16 *Pelagibacter* (SAR11), 4 *Pseudothioglobus* (SUP05), 2 SAR116, and 4 *Njordibacter*. These cultures and closed genomes enhance the understanding of microbial diversity in the Arctic and the evolutionary processes underlying adaptation.

## ANNOUNCEMENT

The Arctic Ocean is a dynamic marine environment characterized by extreme spatial and temporal variation (1–3). It is also warming at four times the global average, threatening habitat and biodiversity (4–7). Environmental sequencing has provided valuable insights into diversity, but natural genomic variation has limited efforts to assemble complete genomes for some of the most abundant lineages (8). While some Arctic species have been cultured and sequenced (9–11), additional cultures and genomes are needed to identify the extent of microbial diversity in the Arctic. Here we report the complete genome sequences from 34 marine bacteria cultured from the Arctic Ocean.

Full methods, genome analyses, and descriptions of family, genus, and species names are described elsewhere (12). Briefly, seawater was collected in May 2023 from the Fram Strait (81.01° N, 10.62° E) through ~2 m thick sea ice. Samples for cultivation were cryopreserved by amending to 10% (vol/vol) glycerol, flash freezing in liquid nitrogen, and stored at −80°C. Cultivation was conducted on cryopreserved samples as previously described (13) with modifications. Cells were diluted to extinction in sterile acid-washed 96-well Teflon plates containing Arctic seawater that was filter sterilized using a 30 kDa Biomax PelliconXL (MilliporeSigma, Burlington, MA). Plates were incubated at 4°C for 13 weeks and monitored for growth using a Guava easyCyte flow cytometer (Cytek, Fremont, CA). Select cultures were grown in 1 L filter sterilized Puget Sound seawater at 4°C for 2–8 weeks and collected onto a 47 mm, 0.2 µm pore size Isopore membrane filter (MilliporeSigma, Burlington, MA) when they reached maximum cell densities (10^5^–10^6^ cells/mL). Cells were lysed by shearing filters into a 2 mL DNA LoBind tube (Eppendorf, Hamburg, Germany) with 200 µL sterile TE and subjected to one −80°C/95°C freeze–thaw cycle. High molecular weight DNA was then extracted using the AutoGen QuickGene DNA Tissue Kit (AutoGen, Holliston, MA) using doubled reagent volumes and a 200 µL elution in molecular grade water. DNA was purified with a 1× (vol/vol) magnetic bead cleanup (Sergi Lab Supplies, Seattle, WA) with two 80% ethanol washes. Cleaned DNA was eluted in 20 µL of molecular grade water.

Whole genome sequencing for each culture was performed on purified genomic DNA using Oxford Nanopore Technologies R10.4.1 Flongle flow cells with the SQK-RAD114 Library Prep Kit (Oxford Nanopore Technologies, Oxford, United Kingdom). Bases were called using Dorado (github.com/nanoporetech/dorado) with the dna_r10.4.1_e8.2_400bps_hac@v4.2.0 model. Reads ≥Q9 and ≥200 bp were assembled using Flye v2.9.1 (14) and polished with Medaka v1.7.2 (github.com/nanoporetech/medaka) on the UseGalaxy web platform (15). Genome annotation was completed by NCBI with the Prokaryotic Genome Annotation Pipeline v6.8 (16). Seven genomes were obtained from cultures containing two species and were given the suffix “_01” or “_02.” All assemblies produced single circular contigs and were reoriented to start with DnaA. Assembly and genome metrics for each genome are outlined in Table 1.

**TABLE 1:** Assembly data of 34 Arctic marine bacteria

Isolate	Lineage	Genome length (Mbp)	Average coverage (fold)	Read length N50 (bp)	GC (%)	Genome accession	Sequence read archive
uisw_002	*Njordibacter*	2.44	200	7,790	44	CP159832	SRR29769124
uisw_016	*Pseudothioglobus* (SUP05)	1.75	79	8,053	36	CP159806	SRR29769128
uisw_017	*Patiriisocius*	3.79	68	10,361	35	CP159835	SRR29769151
uisw_031	*Halioglobus* (OM60)	3.71	121	10,564	52	CP159826	SRR29769150
uisw_039	*Njordibacter*	2.98	274	5,946	45	CP159831	SRR29769123
uisw_041	*Pseudothioglobus* (SUP05)	1.74	34	9,366	37	CP159805	SRR29769127
uisw_047	*Patiriisocius*	2.92	94	10,313	37	CP159834	SRR29769140
uisw_050_01	*Pseudothioglobus* (SUP05)	1.77	167	8,664	36	CP159804	SRR29769126
uisw_050_02	*Porticoccus*	1.44	25	4,987	42	CP159825	SRR29769149
uisw_056	*Njordibacter*	2.98	75	7,523	45	CP159830	SRR29769122
uisw_058	*Njordibacter*	3.00	37	9,051	45	CP159829	SRR29769121
uisw_064	*Marifrigoribacter*	2.86	220	10,979	32	CP159833	SRR29769129
uisw_065	*Aqulinua*	1.39	74	13,077	52	CP159836	SRR29769152
uisw_086	*Pseudothioglobus* (SUP05)	1.77	352	9,355	36	CP159803	SRR29769125
uisw_090	*Pelagibacter* (SAR11)	1.38	16	11,474	30	CP159810	SRR29769133
uisw_092	*Pelagibacter* (SAR11)	1.30	144	13,711	29	CP159824	SRR29769148
uisw_094	*Pelagibacter* (SAR11)	1.35	44	7,093	30	CP159823	SRR29769147
uisw_097	*Methylophilaceae* (OM43)	1.37	260	10,503	35	CP159828	SRR29769120
uisw_099_01	*Methylophilaceae* (OM43)	1.36	33	13,205	34	CP159827	SRR29769119
uisw_099_02	*Pelagibacter* (SAR11)	1.29	86	13,262	29	CP159822	SRR29769146
uisw_101	*Pelagibacter* (SAR11)	1.45	104	11,240	30	CP159821	SRR29769145
uisw_104	*Pelagibacter* (SAR11)	1.44	35	12,461	30	CP159820	SRR29769144
uisw_106	*Pelagibacter* (SAR11)	1.38	40	13,874	30	CP159819	SRR29769143
uisw_113	*Pelagibacter* (SAR11)	1.36	97	11,617	30	CP159809	SRR29769132
uisw_114	*Pelagibacter* (SAR11)	1.39	11	7,316	30	CP159818	SRR29769142
uisw_116	*Pelagibacter* (SAR11)	1.30	20	6,972	29	CP159817	SRR29769141
uisw_121	*Pelagibacter* (SAR11)	1.35	86	7,724	30	CP159816	SRR29769139
uisw_127	*Pelagibacter* (SAR11)	1.33	59	17,865	30	CP159815	SRR29769138
uisw_130	*Pelagibacter* (SAR11)	1.42	252	11,821	29	CP159814	SRR29769137
uisw_134_01	*Levibacter* (SAR116)	2.22	26	11,277	31	CP159808	SRR29769131
uisw_134_02	*Pelagibacter* (SAR11)	1.30	66	10,896	29	CP159813	SRR29769136
uisw_136	*Pelagibacter* (SAR11)	1.39	58	6,622	29	CP159812	SRR29769135
uisw_137	*Pelagibacter* (SAR11)	1.40	50	10,246	29	CP159811	SRR29769134
uisw_141_02	*Ponderosibacter* (SAR116)	2.40	23	8,635	50	CP159807	SRR29769130

## Data Availability

Genome sequences were deposited in GenBank under Bioproject accession number PRJNA1129510. Genome accession and sequence reach archive identifiers for each genome are available in Table 1.
